# Cholesterol-induced mammary tumorigenesis is enhanced by adiponectin deficiency: role of LDL receptor upregulation

**DOI:** 10.18632/oncotarget.1364

**Published:** 2013-10-01

**Authors:** Jing Liu, Aimin Xu, Karen Siu-Ling Lam, Nai-Sum Wong, Jie Chen, Peter R Shepherd, Yu Wang

**Affiliations:** ^1^ Department of Pharmacology and Pharmacy, University of Hong Kong, Hong Kong, China; ^2^ Department of Medicine, University of Hong Kong, Hong Kong, China; ^3^ Research Center of Heart, Brain, Hormone, and Healthy Aging, University of Hong Kong, Hong Kong, China; ^4^ Department of Biochemistry, University of Hong Kong, Hong Kong, China; ^5^ Maurice Wilkins Centre for Molecular Biodiscovery, University of Auckland, Auckland, New Zealand

**Keywords:** Adiponectin, breast cancer, autophagy, cholesterol, LDLR

## Abstract

Adiponectin is an adipokine that can suppress the proliferation of various human carcinoma cells. Although its anti-tumor activities have been suggested by many clinical investigations and animal studies, the underlying mechanisms are not fully characterized. In MMTV-polyomavirus middle T antigen (MMTV-PyVT) transgenic mice models, reduced- or complete loss-of-adiponectin expression promotes mammary tumor development. The present study demonstrated that while tumor development in control MMTV-PyVT mice is associated with a progressively decreased circulating cholesterol concentration, adiponectin deficient MMTV-PyVT mice showed significantly elevated total- and low density lipoprotein (LDL)-cholesterol levels. Cholesterol contents in tumors derived from adiponectin deficient mice were dramatically augmented. High fat high cholesterol diet further accelerated the tumor development in adiponectin deficient PyVT mice. The protein levels of LDL receptor (LDLR) were found to be upregulated in adiponectin-deficient tumor cells. In human breast carcinoma cells, treatment with LDL-cholesterol or overexpressing LDLR elevates nuclear beta-catenin activity and facilitates tumor cell proliferation. On the other hand, adiponectin decreased LDLR protein expression in breast cancer cells and inhibited LDL-cholesterol-induced tumor cell proliferation. Both *in vivo* and *in vitro* evidence demonstrated a stimulatory effect of adiponectin on autophagy process, which mediated the down-regulation of LDLR. Adiponectin-induced reduction of LDLR was blocked by treatment with a specific inhibitor of autophagy, 3-methyladenine. In conclusion, the study demonstrates that adiponectin elicits tumor suppressive effects by modulating cholesterol homeostasis and LDLR expression in breast cancer cells, which is at least in part attributed to its role in promoting autophagic flux.

## INTRODUCTION

The relationship between dysregulated metabolism and carcinogenesis was first enunciated by Otto Warburg more than 80 years ago [1]. There is now a large body of evidence supporting a link between obesity, metabolic syndrome, insulin resistance with increased risk of cancers [2-4]. For example, overweight and obesity account for over 25% of the patients with breast cancer, the most frequent cancer and the second leading cause of cancer death among women [5, 6]. Excess adiposity is associated with late-stage disease and poor prognosis in breast cancer. On the other hand, information is limited on the detailed molecular links between aberrant metabolism in obesity and elevated cancer risks.

Adipokines are a family of molecules selectively secreted by fat tissue. Evidence from clinical, epidemiological and experimental studies suggests that adipokines are key pathological mediators in obesity-related cancer diseases [7-9]. Under obese condition, adipose tissue becomes “inflamed” to produce excess amount of inflammatory adipokines, such as tumor necrosis factor alpha (TNFα), leptin and lipocalin-2, which promote breast cancer cell survival, proliferation and tumor development [3, 10]. On the other hand, the circulating level of adiponectin, the anti-inflammatory adipokine, is inversely associated with obesity-related cancer diseases [11, 12]. Low serum adiponectin levels in obese women are associated with an increased risk for breast cancer development and mortality [13, 14]. Women with higher adiponectin levels have a reduced risk of breast cancer [14, 15]. Moreover, tumors in women with the low serum adiponectin levels are more likely to show a biologically aggressive phenotype with poor prognosis [16, 17]. Numerous experimental studies have shown the anti-proliferative activity of adiponectin in breast cancer cells and animal models [11, 18-22].

In mouse models with spontaneous mammary tumor development, complete loss or insufficient production of adiponectin from adipocyte *per se* promotes mammary tumor onset and development [20, 21]. The present study demonstrates that adiponectin deficiency adversely affects lipid metabolism during tumorigenesis in MMTV-PyVT mice. Elevated circulating cholesterol levels promote mammary tumor development. Adiponectin inhibits cholesterol-stimulated proliferation of mammary tumor cells by reducing the low density lipoprotein receptor (LDLR) expression and cholesterol uptake. These actions of adiponectin are attributed in part to its role in regulating the autophagy process of the breast cancer cells.

## RESULTS

### Accelerated tumor development in adiponectin deficient MMTV-PyVT mice is associated with elevated circulating and tumor cholesterol contents

**Figure 1:**
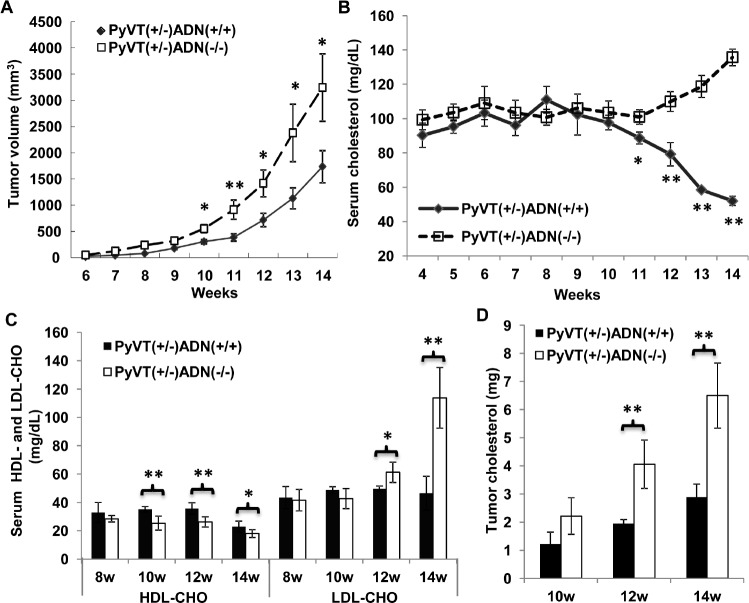
Adiponectin deficiency accelerated breast cancer development and increased serum as well as tumor cholesterol levels in MMTV-PyVT mice Female mice with genotypes of PyVT(+/−)ADN(+/+), PyVT(+/−)ADN(−/−), PyVT(−/−)ADN(+/+) and PyVT(−/−)ADN(−/−) were fed with a standard chow from 3 weeks to 14 weeks. Tumor development (A) was monitored twice a week. Tumor sizes were measured using vernier calipers and tumor volume was calculated as described in Methods. Blood samples were collected from the tail vein of the mice once a week. Serum cholesterol (week 4-14) (B) and HDL/LDL-cholesterol contents (week 8, 10, 12 and 14) (C) were detected from these serum samples. Lipids were extracted from tumor tissues of the PyVT(+/−)ADN(+/+) and PyVT(+/−)ADN(−/−) mice at 10, 12 and 14 weeks. Total cholesterol levels (D) were detected from the tumor lipids. *, p < 0.05, **, p < 0.01, each group contained 10-16 mice, and results were presented as mean ± SD.

Adiponectin deficient MMTV-PyVT mice were generated by backcrossing the original MMTV-PyVT mice with AKO mice in FVB/N background. The litters with control [PyVT(+/−)ADN(+/+)] or deficient adiponectin alleles [PyVT(+/−)ADN(−/−)] were used in the present study. Tumor development was monitored twice a week. From the age of 10 weeks, tumor growth was significantly accelerated in adiponectin deficient mice (Figure 1A). At the age of 14 weeks, the tumor size of PyVT(+/−)ADN(−/−) mice was larger than PyVT(+/−)ADN(+/+) mice by ~1.87 folds. At the time of sacrifice, the total wet weights of tumors were 3.1250 ± 1.4005 g and 1.7512 ± 0.4183 g, respectively, in the two groups of animals. Histological analysis revealed a markedly elevated necrotic area and stromal lymphocytic response in tumors derived from adiponectin deficient PyVT mice (Supplementary Figure 1).

Total serum cholesterol was measured using blood samples collected from mouse tail vein. The results demonstrated that from week 11, serum cholesterol levels in PyVT(+/−)ADN(+/+) mice were progressively decreased, whereas those in PyVT(+/−)ADN(−/−) mice were elevated (Figure 1B). At week 14, the difference between the two groups of mice was ~2.61 folds. Further analysis revealed that the high-density lipoprotein cholesterol (HDL-CHO) levels were reduced by ~35% and ~29% in 14-week old PyVT(+/−)ADN(+/+) and PyVT(+/−)ADN(−/−) mice, respectively, when compared to those at the age of 10 weeks. The low-density lipoprotein cholesterol (LDL-CHO) levels were significantly augmented only in PyVT(+/−)ADN(−/−) mice (Figure 1C). At week 14, the LDL-CHO level in PyVT(+/−)ADN(−/−) mice was increased to nearly two folds of that in PyVT(+/−)ADN(+/+) mice (Figure 1C). These phenomena were not observed in mice carrying no PyVT transgene, irrespective of the adiponectin allele status (data not shown). Next, the cholesterol contents in tumors were evaluated. While at the age of 10 weeks, tumor cholesterol contents were not different between PyVT(+/−)ADN(+/+) and PyVT(+/−)ADN(−/−) mice, those in 12- and 14-week old PyVT(+/−)ADN(−/−) mice were significantly higher. The total amounts of cholesterol in tumor tissues collected from PyVT(+/−)ADN(+/+) and PyVT(+/−)ADN(−/−) mice were 2.89 ± 0.46 mg and 6.50 ± 1.16 mg, respectively (Figure 1D).

### Cholesterol treatment promoted mammary tumor development and breast cancer cell proliferation

**Figure 2:**
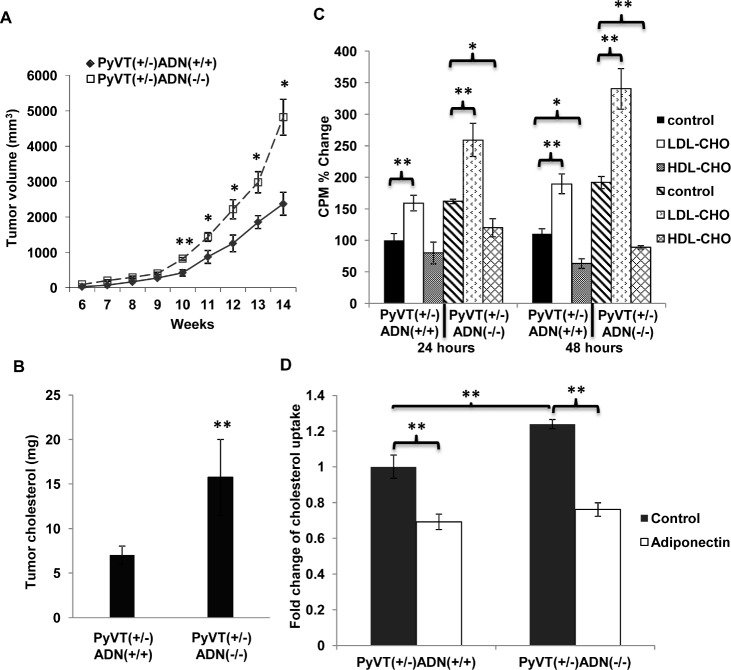
Cholesterol promoted mammary tumor development and breast cancer cell proliferation Female mice were fed with high fat high cholesterol (HFHC) diet from week 3 to week 14. Tumor development (A) was monitored twice a week, n = 15. Lipids were extracted from tumor tissues of the PyVT(+/−)ADN(+/+) and PyVT(+/−)ADN(−/−) mice at 14 weeks under HFHC diet. Total cholesterol levels (B) were detected from the tumor lipids, n = 9. Primary tumor cells were isolated from the PyVT(+/−)ADN(+/+) and PyVT(+/−)ADN(−/−) mice at 14 weeks under standard chow and cultured in serum free DMEM medium containing LDL-CHO (50 μg/ml) or HDL-CHO (50 μg/ml) for 24 hours and 48 hours. Cell proliferation was evaluated using [^3^H]-thymidine incorporation assay (C), n = 4. [^3^H]-cholesterol (10 nM) uptake (D) was performed in these primary tumor cells treated with or without adiponectin (15 μg/ml) in the serum free DMEM medium for 15 min after fasting the cells for 24 hours, n = 6. *, p < 0.05; **, p < 0.01 and the mean value ± SD was presented.

The effect of high fat high cholesterol (HFHC) diet on tumor development was tested in PyVT(+/−)ADN(+/+) and PyVT(+/−)ADN(−/−) mice. The diet treatment significantly reduced the tumor latency in PyVT(+/−)ADN(−/−) mice, for which the tumor onset was recorded at ~42 days, but did not significantly change that of PyVT(+/−)ADN(+/+) mice (~52 days). In both types of mice, tumor development was accelerated by HFHC diet (Figure 2A). Tumors collected at week 14 were much heavier in PyVT(+/−)ADN(−/−) mice (5.1418 ± 1.6334 g) compared to PyVT(+/−)ADN(+/+) mice (2.9562 ± 1.4290 g). Again, the tumor cholesterol content in adiponectin deficient tumor was found to be much higher (15.75 ± 4.25) than that (7.02 ± 1.02) of ADN(+/+) mice (Figure 2B).

The effect of cholesterol on the growth of primary tumor cells isolated from PyVT(+/−)ADN(+/+) and PyVT(+/−)ADN(−/−) mice was evaluated by [^3^H]-thymidine incorporation assay (Figure 2C). The results showed an opposite outcome for LDL-CHO and HDL-CHO treatments. After 48 hours of treatment, LDL-CHO enhanced DNA incorporation by ~58% and ~78% in PyVT(+/−)ADN(+/+) and PyVT(+/−)ADN(−/−) tumor cells, respectively. By contrast, HDL-CHO suppressed cell growth by ~68% and ~76% in these two types of tumor cells (Figure 2C). The results revealed that [^3^H]-cholesterol uptake was increased by ~24% in primary tumor cells derived from PyVT(+/−)ADN(−/−) mice when compared to that of PyVT(+/−)ADN(+/+) mice (Figure 2D). Adiponectin treatment inhibited cholesterol uptake to a similar level in the two types of tumor cells.

**Figure 3:**
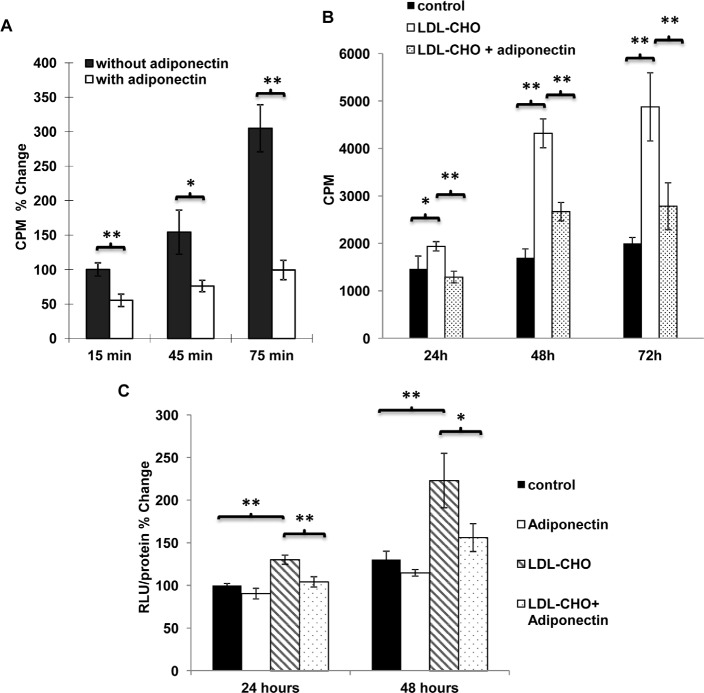
Adiponectin inhibited the uptake of cholesterol and LDL-cholesterol induced cell proliferation as well as β-catenin activity in breast cancer cells *A*, [^3^H]-cholesterol (10 nM) uptake was performed in MDA-MB-231 human breast carcinoma cells treated with or without adiponectin (15 μg/ml) in the serum free DMEM medium for 15, 45 and 75 min after fasting the cells for 24 hours, n = 12. *B*, MDA-MB-231 cells were treated with LDL-CHO (50 μg/ml) or mixtures of LDL-CHO (50 μg/ml) and adiponectin (15 μg/ml) in serum free DMEM medium for 24, 48 and 72 hours. Cell proliferation was evaluated using [^3^H]-thymidine incorporation assay, n = 4. *C*, MDA-MB-231 breast cancer cells were treated with or without LDL-CHO (50 μg/ml) in the presence or absence of adiponectin (15 μg/ml) in serum free medium for 24 and 48 hours for nuclear β-catenin activity detection by TOPflash/FOPflash reporter assay, n = 4. *, p < 0.05; **, p < 0.01 and the mean value ± SD was presented.

[^3^H]-cholesterol uptake in human breast carcinoma MDA-MB-231 cells was also investigated. Adiponectin treatment suppressed [^3^H]-cholesterol uptake and accumulation (Figure 3A). Moreover, in the presence of this protein, the proliferation-stimulatory effects of LDL-CHO were significantly attenuated (Figure 3B). Chronic treatment of adiponectin could modulate beta-catenin activity in MDA-MB-231 cells [11]. Here, the results from TOPflash/FOPflash reporter assay demonstrated that cells cultured in LDL-CHO-containing media showed elevated nuclear beta-catenin activity, which was inhibited by adiponectin treatment (Figure 3C). Note that the hyperactivated beta-catenin signalling was also detected in cells derived from PyVT(+/−)ADN(−/−) mice (Supplementary Figure 2). The nuclear beta-catenin activity was ~2.52 folds higher in their cells compared with that in PyVT(+/−)ADN(+/+) tumor cells.

### Low-density lipoprotein receptor (LDLR) expression is up-regulated in PyVT(+/−)ADN(−/−) tumor cells and promotes breast cancer cell proliferation

**Figure 4:**
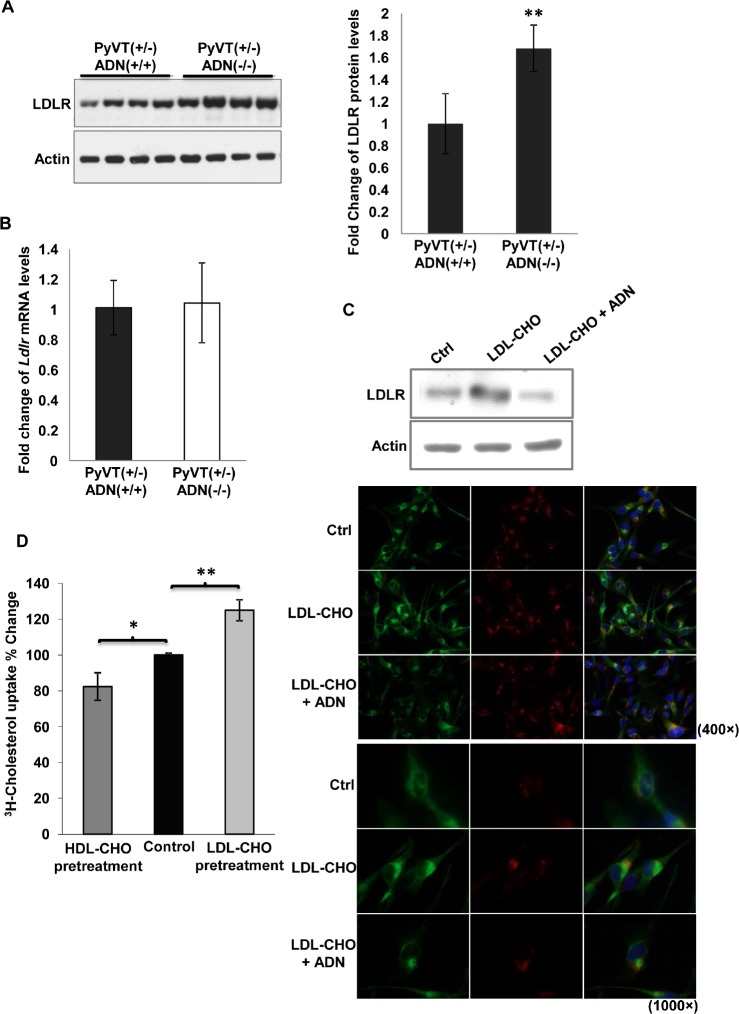
Adiponetin inhibited LDLR expression *A*, The amount of LDLR and actin were detected by Western blotting using the primary tumor cell lysates (left panel). The protein band intensities were quantified by densitometry. Fold changes were calculated and presented in the right panel, n = 4. *B*, mRNA of *LDLR* was detected by QPCR using RNAs derived from primary tumor cells, n = 6. *C*, MDA-MB-231 cells were treated with LDL-CHO (50 μg/ml) or the mixture of LDL-CHO (50 μg/ml) and adiponectin (15 μg/ml) in the serum free medium for 24 h. The expressions of LDLR were detected by Western blotting (upper panel) and immunocytochemistry staining (lower panel). *D*, MDA-MB-231 human breast cancer cells were pretreated with HDL-CHO (50 μg/ml) or LDL-CHO (50 μg/ml) in serum free medium for 24 hours. [^3^H]-cholesterol uptake was measured in these cells after treatment for 15 minutes, n = 4. *, p < 0.05; **, p < 0.01, and the mean value ± SD was presented.

Since surface-expressed LDLR is responsible for cellular uptake of circulating LDL [26], Western blotting was performed to evaluate LDLR expression in primary tumor cells isolated from PyVT(+/−)ADN(+/+) and PyVT(+/−)ADN(−/−) mice. The total amount of LDLR in PyVT(+/−)ADN(−/−) tumor cells was significantly greater than that in PyVT(+/−)ADN(+/+) tumor cells (Figure 4A). The mRNA expression of *Ldlr* was not different between these two types of cells (Figure 4B). In MDA-MB-231 cells, treatment with LDL-CHO induced a sharp elevation of the LDLR protein expression, whereas co-incubation with adiponectin blocked this effect (Figure 4C), and enhanced the cholesterol uptake (Figure 4D).

**Figure 5:**
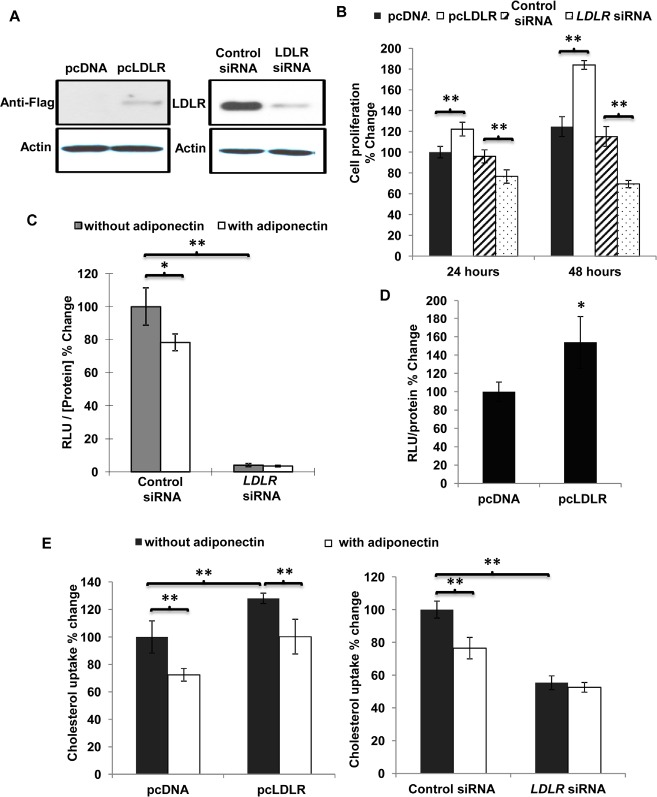
Overexpression of LDLR facilitated cell proliferation, increased cholesterol uptake and activated nuclear β-catenin activity in MDA-MB-231 human breast cancer cells MDA-MB-231 human breast cancer cells were transfected with pcDNA, pcLDLR, control siRNA and *LDLR* siRNA with lipofectamine 2000. *A*, Overexpression of the LDLR was confirmed by Western blotting using anti-flag antibody. Downregulation of LDLR was detected by Western blotting using anti-LDLR antibody. *B*, Cell proliferation was evaluated at 24, 48 hours post-transfection using [^3^H]-thymidine incorporation assay, n = 6. *C*, Cells were transient transfected the *LDLR* siRNA and control siRNA for 24 hours and then treated with or without adiponectin (15 μg/ml) in 10% FBS DMEM for another 24 hours. Nuclear β-catenin activity was analyzed by TOPflash/FOPflash reporter assay, n = 4. Cells were transfected with pcDNA or pcLDLR for 48 hours for nuclear β-catenin activity analysis by TOPflash/FOPflash reporter assay (D), n = 4. *E*, [^3^H]-cholesterol uptake was detected in cells treated with or without adiponectin (15 μg/ml) in 10% FBS DMEM after transient transfection with pcLDLR or pcDNA (left panel) as well as *LDLR* siRNA and control siRNA (right panel), n = 6. *, p < 0.05; **, p < 0.01, and the mean value ± SD was presented.

To evaluate the effect of LDLR on breast cancer cell proliferation, MDA-MB-231 cells were transiently transfected with an expression vector encoding LDLR (pcLDLR) or the specific siRNA targeting *LDLR* (Figure 5A). Compared to that of pcDNA-transfected cells, the proliferation rate in cells over-expressing LDLR was significantly higher and the cell number was increased by ~22% and ~48% after 24 and 48 hours of culture, respectively (Figure 5B). Down-regulation of LDLR expression by transfecting specific siRNA reduced the cell proliferation by ~20% and ~40% compared to cells transfected with control siRNA, at 24 and 48 hours, respectively. TOPflash/FOPflash reporter assay was performed to investigate the effect of LDLR on nuclear beta-catenin activity. Suppression of LDLR expression dramatically reduced the nuclear beta-catenin activity (Figure 5C). Conversely, overexpression of LDLR increased the nuclear activity of beta-catenin compared to MDA-MB-231 cells transfected with control vector (Figure 5D). [^3^H]-cholesterol uptake was increased in cells overexpressing LDLR and dramatically decreased by knocking down the expression of LDLR (Figure 5E). Reducing LDLR expression by RNA interference attenuated cholesterol uptake by over 45% and abolished adiponectin-mediated inhibition of cholesterol uptake in MDA-MB-231 cells.

### Adiponectin promotes LDLR degradation by increasing autophagic flux

**Figure 6:**
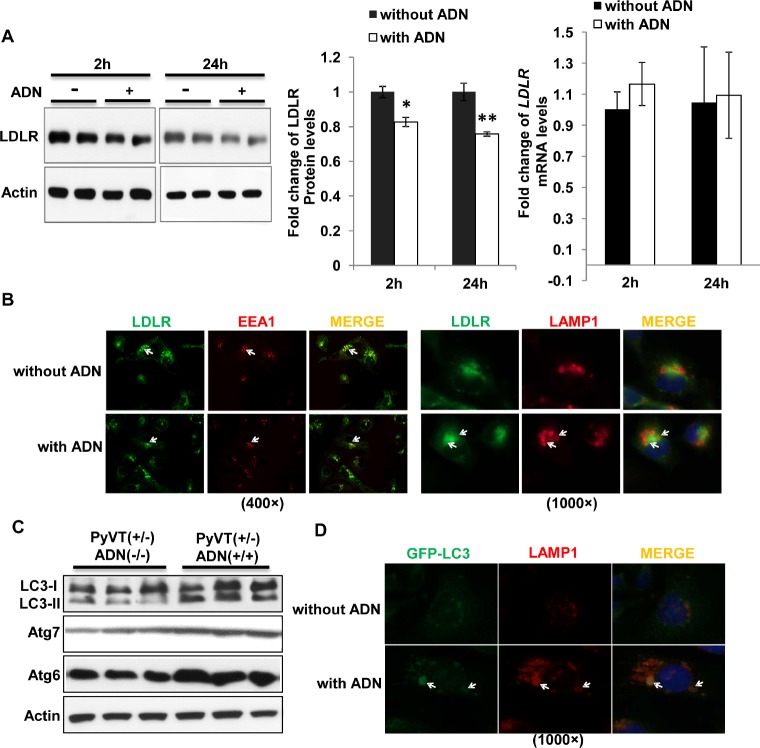
Adiponectin promoted trafficking of the LDLR to lysosome for degradation through increasing autophagosome formation *A*, After fasting for 24 hours in serum free medium, cells were treated with or without adiponectin (15 μg/ml) in the serum free DMEM medium for 2 and 24 hours. LDLR and actin were detected by Western blotting (left panel). Fold changes of the protein band intensities were quantified by densitometry (middle panel). mRNA of *LDLR* was detected by QPCR from MDA-MB-231 cells treated either with or without adiponectin (right panel), n=6-8. *B*, MDA-MB-231 cells were grown on glass coverslips for immunocytochemistry staining. Arrow indicated the co-localization of LDLR and EEA1 as well as LDLR and LAMP1. *C*, Expression of LC3-I, LC3-II, Atg7 and Atg6 were analyzed in the primary tumor cell lysis by Western blotting, n = 3-5. *D*, MDA-MB-231 cells transiently transfected with GFP–LC3 were cultured at 37°C for 24 hours in 10% FBS DMEM medium with or without 15 μg/ml adiponectin and analyzed by immunocytochemistry staining. Arrow indicated the co-localization of LC3-II and LAMP1 in MDA-MB-231 cells treated with adiponectin. *, p < 0.05; **, p < 0.01 and the mean value ± SD was presented.

Both short (2 hours)- and long (24 hours)-term treatments with adiponectin reduced the protein abundance of LDLR in MDA-MB-231 cells, but had no effect on the mRNA expressions (Figure 6A). Immunofluorescent staining demonstrated that adiponectin did not affect the trafficking of LDLR to early endosome, as indicated by the co-localization of LDLR and early endosome antigen (*EEA*)*-*1 in cells treated either with or without this protein. However, compared to vehicle treatment, it promoted the shuttling of LDLR into vesicles containing l*ysosomal-associated membrane protein 1* (*LAMP1*) (Figure 6B).

Autophagy is a process to facilitate the degradation and recycle of intracellular proteins and organelles [27]. The first step of autophagy is the formation of autophagosome to sequester proteins or organelles into a double-membrane structure, which is then fused with lysosome to form autolysosome. An autophagy marker, ubiquitin-like protein Atg8 (known as LC3 in mammalian cells), was detected in primary tumor cells by Western blotting as two bands, LC3-I and LC3-II (Figure 6C). LC3-I is a cytosolic protein, whereas LC3-II is a membrane-binding protein specifically associated with autophagosome and correlated with the extent of autophagosome formation [28]. Expression of LC3-II was decreased in primary tumor cells from PyVT(+/−)ADN(−/−) mice compared to those from PyVT(+/−)ADN(+/+) mice (Figure 6C). Another two autophagy markers, autophagy-related protein 7 (Atg7) and Atg6 (also known as Beclin-1), were also found to be decreased in PyVT(+/−)ADN(−/−) tumor cells. In MDA-MB-231 cells transiently transfected with a plasmid expressing GFP-LC3, adiponectin treatment not only increased the number of puncta per cell, which represented the formation of autophagosomes, but also promoted the formation of autolysosomes as indicated by the large vacuoles containing both GFP-LC3 and LAMP1 (Figure 6D).

**Figure 7:**
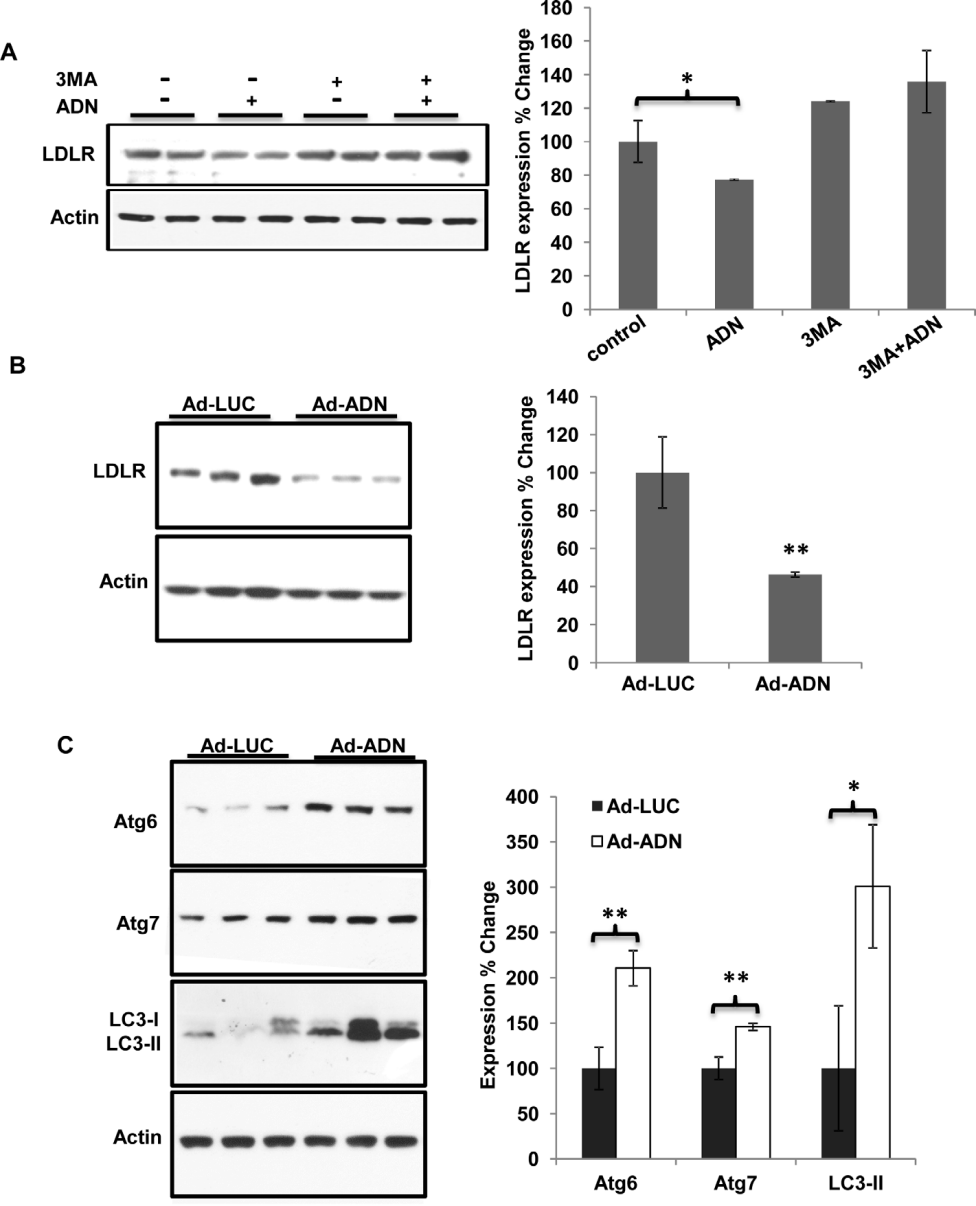
Adiponectin promoted LDLR degradation by increasing autophagy *A*, MDA-MB-231 cells were treated with 3-MA (8 mM) and/or adiponectin (15 μg/ml) in 10% FBS DMEM for 24 hours. Expression of LDLR was detected by Western blot (left panel). Fold changes of protein band intensities were quantified by densitometry (right panel), n = 3. *B*, MDA-MB-231 cells (5×10^6^) were injected into the right thoracic mammary fat pad of 6-week-old female nude mice under anesthetic condition. The recombinant adenovirus [10^8^ plaque-forming units (pfu)] that expressed adiponectin (Ad-ADN) or luciferase (Ad-LUC) was injected into the tumor of each mouse at the 14^th^ day after initial implantation. All mice were sacrificed at 5 weeks after the initial implantation as described before [11]. Expression of LDLR was detected from the tumors by Western blot (left panel) and fold changes of protein band intensities were quantified by densitometry (right panel), n = 3-5. *C*, Expression of Atg6, Atg7 and LC3 were detected from the above tumors derived from nude mice by Western blot (left panel). Fold changes of protein band intensities were quantified by densitometry (right panel), n = 3-5. *, p < 0.05, **, p < 0.01 and the mean value ± SD was presented.

The specific inhibitor of autophagy, 3-methyladenine (*3-MA*), abolished adiponectin-induced reduction of LDLR in MDA-MB-231 cells in culture (Figure 7A). Moreover, in tumors collected from nude mice orthotopically implanted with MDA-MB-231 cells, adiponectin over-expression reduced the LDLR protein expression (Figure 7B). The stimulatory effects of adiponectin on autophagy markers, Atg7, Atg6 and LC3, could also be observed (Figure 7C).

## DISCUSSION

Evidences from epidemiologic studies have suggested that lower circulating adiponectin levels are associated with an increased risk of breast cancer in postmenopausal women [14]. The association can be found in women regardless of menopausal status [15, 29]. Moreover, tumors in women with low serum adiponectin levels are more likely to show biologically aggressive phenotypes and poor prognosis [15], such as large size of tumor and high histological grade.

MMTV-polyomavirus middle T antigen (PyVT) transgenic mice, a well characterized breast cancer mouse model, were used in this study. These transgenic mice express high levels of the transforming oncogene PyVT under the control of the mouse mammary tumor virus promoter, which specifically directs expression to the mammary epithelium [30]. All female PyVT mice spontaneously develop multifocal adenocarcinomas in the mammary gland, with dysplastic foci occurring as early as age 7 weeks. Importantly, this mouse model recapitulates human breast cancer progression from early hyperplasia to malignant breast carcinoma [31, 32]. In the present study, the MMTV-PyVT transgenic mouse models under the condition of deficient adiponectin expression [ADN(−/−)] were established. Adiponectin deficiency accelerated the tumor development compared with control [ADN(+/+)] mice (Figure 1). Histological analysis of the tumor demonstrated markedly elevated tumor necrosis and stromal lymphocytic response in the tumors from ADN(−/−) mice (Supplementary Figure 1), which was consistent with our previous results found in PyVT transgenic mice with reduced adiponectin expression [20]. Adiponectin insufficiency might result in the development of a basal-like subtype tumor, which developed tumor more aggressively [20]. Breast cancers of the basal-like subtype comprise 19% of the tumors and have poor clinical outcomes, which likely reflect this subtype's high proliferative capacity as well as the lack of directed therapies since basal-like tumors do not typically express ER or HER2 [33]. It was in accordance with the clinical evidence that adiponectin deficiency might result in the development of a tumor, which was more aggressive and had poor prognosis [14, 15]. Taken together, the above information suggests that PyVT mice with adiponectin deficiency are suitable animal models to study the mechanisms underlying the adiponectin insufficiency-induced breast carcinogenesis.

Epidemiological studies show that diet and obesity represent significant risk factors for cancer development [5, 6]. In this study, high fat high cholesterol diet (HFHC) diets reduced the tumor latency in PyVT(+/−)ADN(−/−) mice. In addition, HFHC promoted tumor development of both kinds of PyVT mice and increased the necrosis in tumors compared with the standard chow group. It was consistent with the results from other groups that obesity induced by high fat diet significantly accelerated tumor growth and shorten tumor latency in tumor-bearing mice [34, 35].

It has been known for about a century that cholesterol and other lipids accumulate in solid tumors [36]. Experimental evidence showed that cholesterol, cholesterol oxidation products (Oxysterols) and cholesterol precursor (mevalonate) enhanced tumorigenesis [37, 38]. In addition, 3-hydroxy-3-methylglutaryl-coenzyme A (HMG-CoA) inhibitors exhibited pleiotropic antineoplastic effects in a variety of tumors [39]. In our study, total plasma cholesterol levels were reduced in the PyVT(+/−)ADN(+/+) mice during tumorigenesis (Figure 1B), which was similar with the clinical studies. Evidence from epidemiologic and experimental studies have documented an association between low circulating cholesterol and high overall cancer incidence and mortality in prostate cancer [40], breast cancer [37] and several other cancers [41, 42]. This correlation might attribute to the increased utilization of cholesterol by tumors. Our *in vitro* studies showed that LDL-CHO promoted nuclear beta-catenin activity and facilitated proliferation of breast cancer cells or primary cancer cells isolated from the PyVT mice (Figure 3). Adiponectin deficiency elevated plasma cholesterol and LDL-CHO levels (Figure 1), which could further enhance the breast cancer development and activate AKT/GSK3beta/beta-catenin signalling pathway, resulting in accelerating tumorigenesis. This was further corroborated by the cholesterol contents in the tumors showing higher cholesterol contents in tumors from PyVT(+/−)ADN(−/−) mice than those in the PyVT(+/−)ADN(+/+) mice (Figure 1&2).

Increasing cholesterol levels in tumor tissues have been attributed to multiple mechanisms, including increased absorption from the circulation, loss of feedback regulation through downregulation of low-density lipoprotein receptors, and upregulation of components of the mevalonate pathway, particularly HMG-CoA reductase [43, 44]. Cells, as in other tissues, synthesize cholesterol endogenously via the mevalonate pathway. Cellular cholesterol also derives from absorption from circulating lipoproteins. Control of cellular cholesterol content is a balance between metabolic processes intrinsic to the cells and the regulation of cholesterol distribution by the organism. Low-density lipoprotein receptor (LDLR), a plasma cholesterol homeostasis regulator, plays an important role in carcinogenesis. This pathway provides cells with essential fatty acids for prostaglandin E2 (PGE2) synthesis. PGE2 synthesis in prostate cancer cells was significantly increased in response to LDL. Over-production of LDLR is an important mechanism in cancer cells for obtaining more essential fatty acids through LDLR endocytosis, allowing increased synthesis of prostaglandins, which subsequently stimulate cell growth. Over-expression of LDLR occurs in many types of malignancies and is related to the rapid proliferation of tumors. In *adenomatous polyposis coli* (*Apc*)-deficient Min mice, LDLR was overexpressed in the epithelia of the upper part of polyps where lipid droplets were observed. The expression levels of *Ldlr* mRNA in the intestinal polyps of Min mice were approximately three times higher compared to those in the non-tumor parts [45]. The expression level of LDLR was up-regulated in human lung adenocarcinoma A549 cells [46] and colorectal carcinoma cells [47]. In our study over-expression of LDLR facilitated proliferation of breast cancer cells (Figure 5B) and increased stability and nuclear beta-catenin activity (Figure 5D) as well as elevated cholesterol uptake (Figure 5E), thereby promoting tumor development. By contrast, down-regulated LDLR attenuated cell proliferation (Figure 5B) and nuclear beta-catenin activity (Figure 5C) as well as inhibited cholesterol uptake (Figure 5E) in MDA-MB-231 human breast cancer cells.

An interesting phenomenon in this study is that adiponectin deficiency increased total cholesterol and LDL-CHO levels compared with the ADN(+/+) mice during tumor development (Figure 1), which might facilitate the uptake and utilization of cholesterol by tumors, accelerate the tumor development and exacerbate their aggressiveness. There are discrepancies in the two kinds of mice during tumor development. In ADN(+/+) mice at the later stage of tumor development, adiponectin level decreased and cholesterol level also decreased; while in ADN(−/−) mice, no adiponectin expression is associated with increased cholesterol levels. The effects of adiponectin on lipid metabolism could explain these phenomena. Low serum adiponectin is commonly associated with dyslipidemia characterized by an increase in plasma levels of triglyceride (TG), LDL-CHO, apolipoprotein B-48, apolipoprotein C-III, chylomicrons and liver fat content, as well as a decrease in whole-body fat oxidation and HDL-CHO, independent of age, sex, body mass index, and diabetes [48, 49]. Here, the altered lipid metabolism was further corroborated by the lipid contents in the livers (Supplementary Figure 3). Adiponectin deficiency increased the fat accumulation in liver. Adiponectin influences plasma lipoprotein levels by altering the levels and activity of key enzymes (lipoprotein lipase and hepatic lipase) responsible for the catabolism of TG-rich lipoproteins and HDL [50]. Thus, the elevated TG, cholesterol and LDL-CHO levels in PyVT(+/−)ADN(−/−) mice might partly be the consequence of altered lipid metabolism in liver under adiponectin deficient condition during tumor development.

Autophagy plays important role both in carcinogenesis and lipid metabolism. It suppresses tumorigenesis at the early stage, while promotes tumor progression at the later stage of tumor development [51]. Moreover, autophagy regulates lipid content. Inhibition of autophagy in cultured hepatocytes and mouse liver increases triglyceride storage in lipid droplets [52]. In our study, adiponectin increased autophagy both *in vitro* (Figure 6) and *in vivo* (Figure 7C). Adiponectin decreased LDLR expression (Figure 4A, 6A & 7B) and trafficked LDLR to lysosome for degradation (Figure 6B) through autophagy (Figure 7A). Increasing levels of TG and cholesterol in livers and serum (Supplementary Figure 3 & Figure 1B) from PyVT(+/−)ADN(−/−) mice compared to those in the PyVT(+/−)ADN(+/+) mice might partially attributed to the inhibition effects of autophagy in adiponectin knockout condition.

In summary adiponectin deficiency increased obesity-associated breast malignancies in a MMTV-PyVT transgenic mouse model. Adiponectin knockout altered the lipid metabolism in the liver, by secretion of more TG, LDL-CHO and cholesterol into the plasma, which further enhance the breast cancer development. In the adiponectin wild type PyVT mice, adiponectin had the direct actions to the cancer cells to attenuate cell proliferation as well as indirect mechanisms regulating systemic lipid metabolism and inhibition of cholesterol accumulation in the tumor by autophagy-mediated down-regulation of LDLR. Therefore, adiponectin has a potential role in cancer prevention and/or therapy.

## MATERIALS AND METHODS

### Materials

Anti-β-catenin, anti-phosphorylated (Ser^473^) AKT, and anti-phosphorylated (Ser^9^) glycogen synthase kinase-3β (GSK-3β), Atg7, Atg6, LC3 antibodies were obtained from Cell Signaling (Beverly, MA). Goat anti-β-actin was obtained from Santa Cruz Biotechnology (Santa Cruz, CA). Anti-LDLR and anti-LAMP1 antibodies were obtained from Abcam (Cambridge, MA). Anti-EEA1 antibody was obtained from BD Tranduction Laboratories. The MDA-MB-231 cells were obtained from the American Type Culture Collection (Manassas, VA). ImProm-II™ Reverse Transcription System and Bright-GloTM luciferase assay system were from Promega (Madison, WI). TOP/FOPflash (T-cell factor-lymphoid enhancer factor-1 (TCF-LEF) reporter plasmid) was from Upstate (Lake Placid, NY). pcDNA 3.1 (+) and pcLDLR were from Invitrogen (Carlsbad, CA) and Fulengen (Guangzhou, P.R. China), respectively. pLC3B-GFP vector was supplied by Dr. NS Wong from the University of Hong Kong. Recombinant adiponectin was produced from *Escherichia coli* as previously described [11]. Low-density-lipoprotein (LDL), High-density-lipoprotein (HDL) and 3-Methyladenine (3-MA) were obtained from Sigma (Saint Louis, MO). [^3^H]-cholesterol solution and [^3^H]-thymidine solution were from Amersham Biosciences (Uppsala, Sweden).

### Establishment of the MMTV-PyVT transgenic mice without adiponectin expression

FVB/N-Tg(MMTV-PyVT)634Mul/J transgenic mice were obtained from the Jackson Laboratory (Bar Harbor, Maine). MMTV-PyVT mice with adiponectin insufficient [PyVT(+/−)ADN(+/−)] were established as described previously [20]. A FVB/N line of adiponectin insufficient mice [PyVT(+/−)ADN(+/−)] was established by backcrossing this female adiponectin insufficient mice [PyVT(−/−)ADN(+/−)] with male PyVT(+/−) mice from Jackson Laboratory (FVB/N background) for at least 18 generations. Since adiponectin knockout PyVT mice [PyVT(+/−)ADN(−/−)] could not be obtained by crossbreeding male PyVT(+/−)ADN(+/−) with female PyVT(−/−)ADN(+/−) [20], the FVB/N line of adiponectin knockout mice [PyVT(−/−)ADN(−/−)] were established by crossing the littermates from the male PyVT(−/−)ADN(+/−) with female PyVT(−/−)ADN(+/−). PyVT(+/−)ADN(−/−) mice were generated from male PyVT(+/−)ADN(+/−) crossed with female PyVT(−/−)ADN(−/−). Female mice were used for all experiments in the present study. Tumor development was closely monitored twice a week. Tumor latency was recorded as the age of mice when palpable tumors were first detected in at least one of the ten mammary fat pads. Tumor size was measured using a vernier caliper and tumor volume was calculated using the formula [sagittal dimension (mm) × cross dimension (mm)^2^] / 2 and expressed in mm^3^. Serum total cholesterol, LDL-cholesterol and HDL-cholesterol were examined according to the manual of individual kits from Standbio Laboratory (TX, USA). All animal experimental protocols were approved by the Animal Ethics Committee at the University of Hong Kong and their care was in accord with the institution guidelines.

### Isolation of primary mammary tumor cells

Primary mammary tumor cells were isolated as described previously with slight modifications [23]. Fresh tumors were collected aseptically from anesthetized PyVT mice, mechanically minced, suspended in serum-free high glucose DMEM medium and passed through 40 μm sterile nylon cell strainers (BD Falcon). Tumor cell suspension was further disintegrated by serial passaging through a 10 ml syringe without a needle. After a brief centrifugation at 1,000 rpm for 5 minutes at room temperature for the removal of cell debris and the low-density stromal cells, pellets predominantly containing tumor cells were suspended in serum free high glucose DMEM medium for viable cell counting using 0.4% trypan blue. For *in vitro* cell culture, isolated primary tumor cells were suspended with high glucose DMEM medium with 10% FBS, cultured and attached overnight in a 37 °C incubator with humidified atmosphere of 5% CO_2_. The culture medium was then changed every other day. Attached cells were subjected to other *in vitro* assays.

### [^3^H]-thymine incorporation assay for cell proliferation measurement

Cells were seeded at a density of 5000 per well in 96-well plates. After starving in DMEM for 24 hours, cells were treated at different conditions as indicated. One μCi/ml of [^3^H]-thymidine was added into each well for the last 6 hours of treatment. At the end of the experiment, the culture media were removed and cells were washed with cold PBS. DNA was precipitated with 0.5% trichloroacetic acid for 30 minutes. Air-dried precipitates were then solubilized with 0.2 M NaOH, neutralized with 0.2 M HCl, and incorporated [^3^H]-thymidine was qualified with a liquid scintillation counter (Backman LS6500).

### Lipid extraction from tissues

Tissue lipids were isolated as described with slight modifications [24]. Total lipids from 100 mg tumor tissues were extracted with an organic solvent mixture of chloroform and methanol (vol:vol 2:1). Tissue debris or insoluble protein was pelleted by centrifugation at 14,000 rpm at 4 °C for 5 minutes. Soluble protein was separated by water extraction. Samples were centrifuged at 12,000 × *g* at 4 °C for 5 minutes to separate lipid with soluble proteins. The lower layer containing lipid was aspirated and dried under nitrogen gas flow. Lipids were emulsified by sonication in a PBS buffer containing 0.1% Triton X-100. Cholesterol was examined according to the manuals of individual kits from Standbio Laboratory (TX, USA).

### Immunocytochemistry staining

MDA-MB-231 human breast cancer cells were trypsinized and subcultured onto the coverslips. After various treatments, the cells were fixed with 4% paraformaldehyde (PFA) in PBS for 15 minutes and permeabilized in PBS with 0.2% Triton X-100 or 0.2% Tween 20 for 5 minutes at room temperature. After three washing steps in PBS, cells were incubated with blocking buffer (3% BSA in PBS) for 1 hour at room temperature. Then cells were incubated with a primary antibody diluted in PBS containing 1% BSA in a humid chamber at 4°C overnight. After washing another three times with PBS, the cells were incubated with the goat anti-rabbit Alexa Fluor 488 and goat anti-mouse Alexa Fluor 594 secondary antibodies (Invitrogen, California, USA) in PBS with 1% BSA in the dark at room temperature for 1 hour. The coverslips were then washed three times with PBS and mounted with ProLong Gold Anti-fade Reagent (Invitrogen, California, USA) for durable visualization. The coverslips were then observed under a fluorescence microscope (Leica Microsystems, Bensheim, Germany), and the images were captured using AxioVision Imaging Plus software (Carl Zeiss, Inc., International).

### TOPflash/FOPflash β-catenin/T-cell factor-lymphoid enhancer factor (TCF-LEF) reporter assay

Nuclear activities of endogenous β-catenin were analyzed by the TOPflash/FOPflash reporter system (Upstate, Charlottesville, VA) as described previously [11]. Cells grown in 24-well plates under different treatments were transfected with either the TOPflash or FOPflash plasmid using lipofectamine reagent (Invitrogen, Carlsbad, CA). To normalize the transfection efficiency in the reporter assays, cells were co-transfected with pRL-TK plasmid, which contains a functional *Renilla* luciferase gene cloned downstream of a herpes simplex virus thymidine kinase promoter (Promega, Madison, WI). At the point of harvest, cells were washed with PBS and lysed with the reporter lysis buffer (Promega). Cell lysates were collected and luciferase activity was measured using Bright-GloTM Luciferase Assay System (Promega, Madison, WI) on Lumat LB9507 (Berthold Technologies, Bad Wildbad, Germany) and normalized against control *Renilla* luciferase signal. Luciferase activity was calculated against the negative control signal and fold changes were compared among groups in separate assays.

### [^3^H]-cholesterol uptake

Cells were seeded at a density of 2×10^4^ per well in 24-well plates. After starving in DMEM for 24 hours, cells were treated at different conditions as indicated. Ten nanomole [^3^H]-cholesterol (Amersham Biosciences, Uppsala, Sweden) was added in serum free DMEM to the cells for 15, 45 and 75 minutes. Prior to isotope addition, the original [^3^H]-cholesterol/toluene suspension was dried down under N_2_ and resuspended in the same volume of 100% ethanol as indicated [25]. By the end of the experiment, the culture media were removed and cells were washed three times with cold PBS. Cells were then lysed with 0.2 M NaOH, neutralized with 0.2 M HCl, and subjected to liquid scintillation counting to determine cellular cholesterol uptake with a liquid scintillation counter (Backman LS6500).

### Quantitative RT-PCR

Total RNA was isolated and used for the synthesis of cDNA. Quantitative RT-PCR was performed using SYBR® Green real-time PCR reagents were from Qiagen (Hilden, Germany). The reactions were carried out on a 7000 Sequence Detection System (Applied Biosystems, Foster City, CA). Quantification was achieved using Ct values that were normalized with *GAPDH* as internal control. The primers for mouse *Ldlr* were (F) 5’-TGACTCAGACgAACAAGGCTG-3’ and (R) 5'-ATCTAGGCAATCTCGGTCTCC-3'. The primers for mouse *Gapdh* were (F) 5'-CAGAACATCATCCCTGCATC-3' and (R) 5'-CTGCTTCACCACCTTCTTGA-3'. The primers for human *LDLR* were (F) 5'-GCTTGTCTGTCACCTGCAAA-3' and (R) 5'-AACTGCCGAGAGATGCACTT-3'. The primers for human *ACTIN* were (F) 5'-TGACCCAGATCATGTTTGAGA-3' and (R) 5'-AGTCCATCACGATGCCAGT-3'.

### Western blotting

Total cell lysates were separated by SDS-PAGE, transferred to polyvinylidene difluoride membranes, and then probed with various primary antibodies to determine the expression of the signaling proteins, including phosphorylated AKT, GSK-3β, total β-catenin, actin and autophagy markers in MDA-MB-231 cells and primary tumor cells. The antibody-antigen complexes were detected using an enhanced chemiluminescence kit from GE Healthcare (Uppsala, Sweden).

### Statistics

All the experiments were repeated for at least three times. For Western blotting, representative images were shown. Results were presented as mean ± standard deviation (SD). Statistical analysis of difference between two groups was performed using Student's *t*-test. A p-value of less than 0.05 represents a significant difference in all statistical comparisons (*, p < 0.05; **, p < 0.01).

### Declaration of interest

The authors declare that there is no conflict of interest that could be perceived as prejudicing the impartiality of the research reported.

## Supplementary Figures


