# Supplementary material for: Cholesterol-induced mammary tumorigenesis is enhanced by adiponectin deficiency: role of LDL receptor upregulation
Source: Oncotarget. 2013 Oct 1;4(10):1804–18. doi: 10.18632/oncotarget.1364 (PMC3858565; doi:10.18632/oncotarget.1364)
Supplement: Supplementary file 1 [file oncotarget-04-1804-s001.pdf]

Cholesterol-induced mammary tumorigenesis is enhanced by adiponectin deficiency: role of LDL receptor upregulation - Liu et al

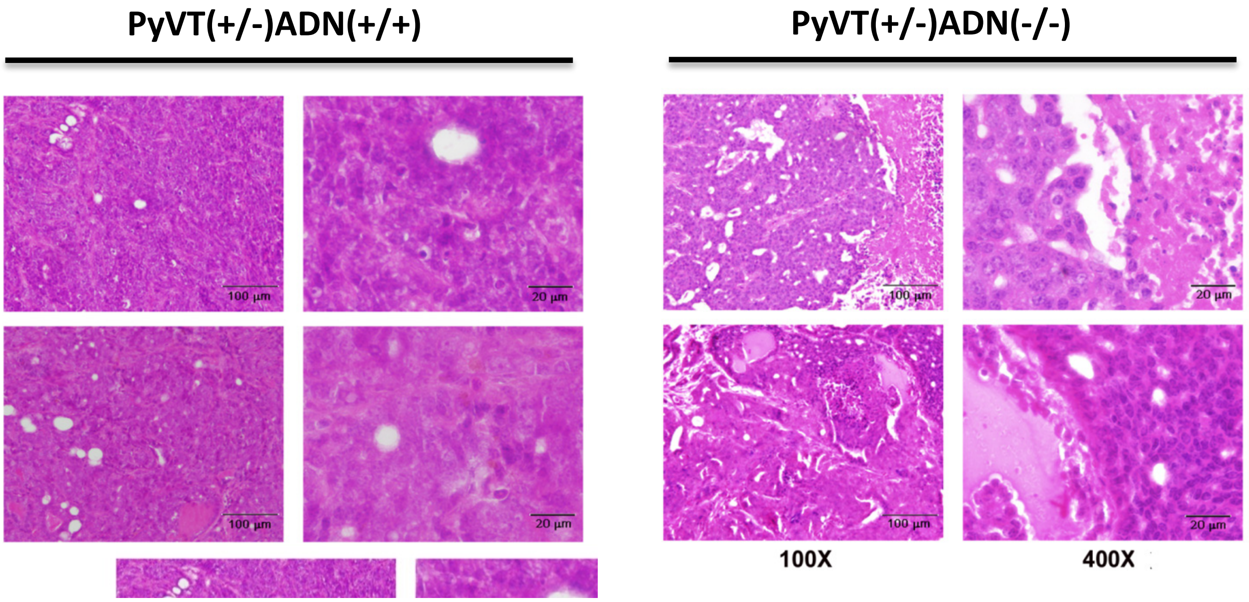

Supplementary Figure 1. Morphological features of mammary tumors derived from 14-week old female PyVT(+/-)ADN(+/+) and PyVT(+/-)ADN(-/-) mice by HE staining. Note that central necrosis and geographic tumor necrosis were found in PyVT(+/-)ADN(-/-) tumors.

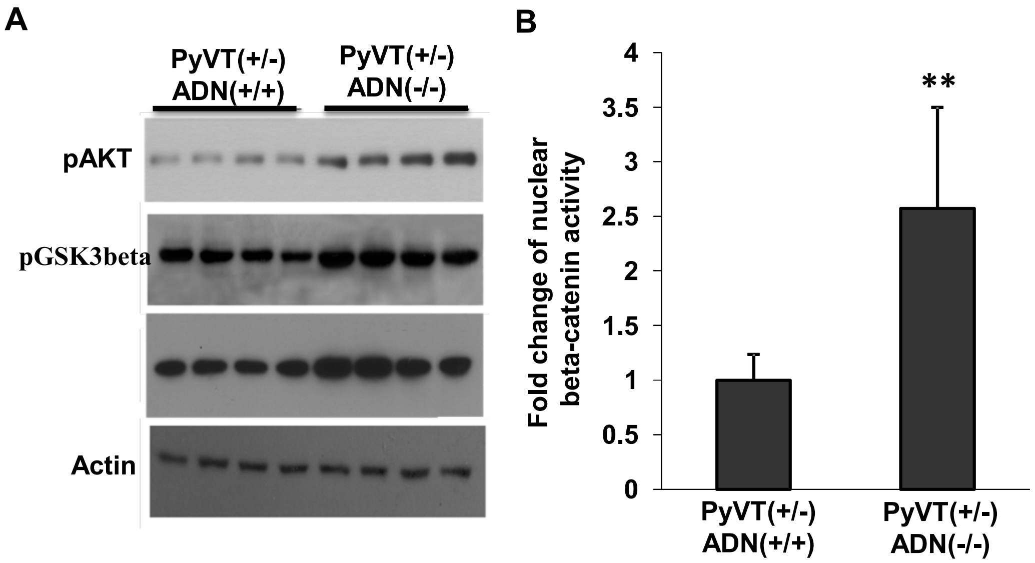

Supplementary Figure 2. Tumor cells derived from PyVT(+/-)ADN(-/-) mice showed hyperactivated AKT/GSK3β/β-catenin signalling. Tumor cells were isolated from PyVT(+/-)ADN(-/-) and PyVT(+/-)ADN(+/+) tumors. Serum-stimulated phosphorylation of AKT, GSK3β and β-catenin were analyzed by Western blotting (A). β-catenin activities (B) were detected using TOPflash/FOPflash reporter assay. **, $p < 0.01$, $n = 6$.

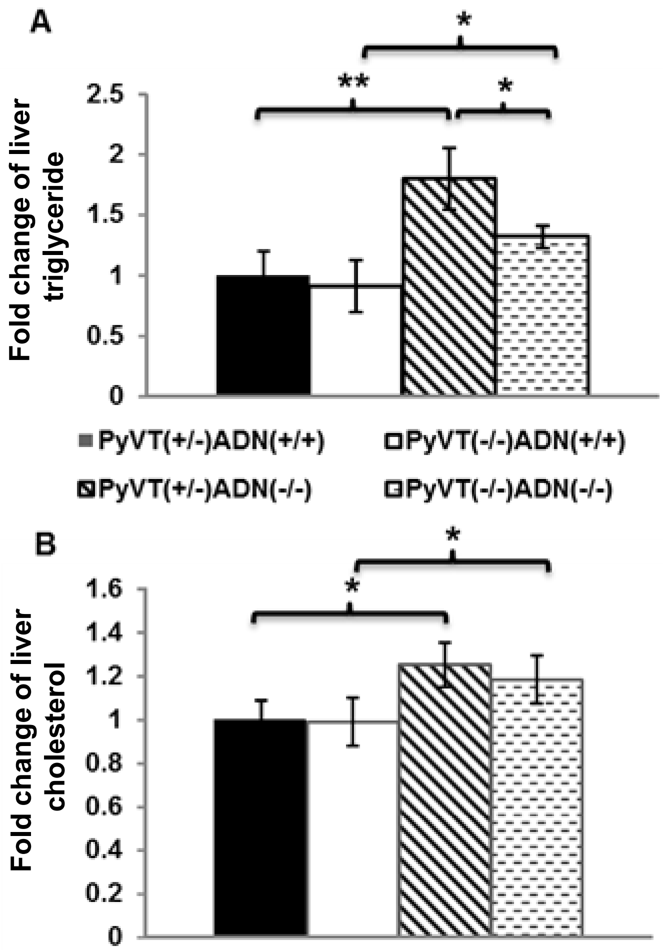

Supplementary Figure 3. Measurement of triglyceride and cholesterol levels in mouse liver tissues. Triglyceride (A) and cholesterol (B) were detected from lipids extracted from the livers. *, $p < 0.05$; **, $p < 0.01$, n = 8-10.
